# What Academic Factors Influence Satisfaction With Clinical Practice in Nursing Students? Regressions vs. fsQCA

**DOI:** 10.3389/fpsyg.2020.585826

**Published:** 2020-12-18

**Authors:** David Fernández-García, María Del Carmen Giménez-Espert, Elena Castellano-Rioja, Vicente Prado-Gascó

**Affiliations:** ^1^Department of Nursing, Catholic University of Valencia, Valencia, Spain; ^2^Department of Nursing, Faculty of Nursing and Chiropody, University of Valencia, Valencia, Spain; ^3^Social Psychology Department, Faculty of Psychology, University of Valencia, Valencia, Spain

**Keywords:** clinical practice, evaluation, graduate education, nursing, student experience, satisfaction

## Abstract

Clinical practices are considered one of the cornerstones in nurses' education. This study provides a framework to determine how factors in the academic environment, influence nursing student's satisfaction with their practices. A cross-sectional analytical study was conducted in a convenience sample of 574 nursing students at a private university in Valencia, during the 2016/2017 academic year, 79% (456) were women. Two statistical methodologies were used for data analysis: hierarchical regression models (HRM) and fuzzy sets qualitative comparative analysis (fsQCA). The HRM indicate that the students' mean score influences all dimensions of satisfaction. Furthermore, in the fsQCA, the type of service and center, as well as the type of management, the preference in the choice of the practice center and the number of students per period per clinical educator influence satisfaction with clinical practices. These results could be used to understand how academic factors influence nursing students' satisfaction with their clinical practices and to create intervention programmes that improve it. This will help prepare students to be the future nursing workforce.

## Introduction

Marketing literature has shown an interest in the relationship between quality management and consumer satisfaction (Rauyruen and Miller, 2007), because perceptions of quality and judgments of satisfaction have been recognized as fundamental aspects in explaining desirable consumer behaviors (Zeithaml et al., 1993). One of the major challenges that higher education institutions face today is improving the quality of the education system (Pucciarelli and Kaplan, 2016). The approaches created since the 1980s, based around the importance of studying the concept of quality of service and its evaluation (Parasuraman et al., 1985; Cronin and Taylor, 1992), have led to a paradigm in which it has been consolidated as a key aspect in the success of any business model (Parasuraman et al., 1988; Cronin and Taylor, 1992). In the field of higher education, interest in this concept has increased as a result of the growth in the number of university students, the interest in improving public services, the increase in competition in the education market and the tension between efficiency and quality (Green, 1994). Advances in the study of the quality of the service have shown a relationship between its dimensions and the satisfaction of the user/consumer (Falk et al., 2010), and with the intention to purchase and later recommendation (Olorunniwo et al., 2006).

Quality of service has been defined in many ways, but one of the most widely accepted definitions is a type of activity that reflects the excellence or superiority of a particular service, placing the emphasis of this quality on the relationship between the employee and the user (Parasuraman et al., 1985). In the educational sphere, it refers to quality in terms of the purpose of the product, i.e., quality must be judged according to whether the product or service offered accomplishes the stated purpose (Green, 1994). Finally, when evaluating quality from the perspective of expectations, it is essential to address the needs of users, by considering subjective factors related to the judgments of the people who receive the product or service (Moreno et al., 2015). The quality of an educational system, a university degree or nursing practices can be measured using many different variables, since many factors affect it, but the satisfaction that this system, this degree or these practices generate among users has long been one of the variables to be taken into account, thus becoming a key element in teaching quality (Löfmark et al., 2012). In addition, in order to provide a quality service, it is necessary to resolve all the aspects related to the functioning of the organization, taking into account that the differences between service expectations and perceptions can be added to others, such as discrepancies in the organization's functioning (García-Pascual et al., 2020).

The perceptions of quality and satisfaction judgments a student may have about their degree course, the institution and their organization have been recognized as key constructs in explaining the behaviors of “consumers” of a product, which in this case is an educational product (Rauyruen and Miller, 2007). In fact, several studies state that the satisfaction created by the educational process is an inherent element of quality (Löfmark et al., 2012), and as such analyzing the students' view of their clinical training can provide very valuable information for reconstructing and improving the teaching activity and the level of nursing education (Papathanasiou et al., 2014). Including student participation and perceptions in the evaluation of the quality of educational programs is therefore essential (Haraldseid et al., 2015), since improving the students' experience in their clinical learning environment can improve students' learning outcomes (Payne, 2016) and satisfaction.

Clinical practices are considered one of the cornerstones in nurses' education. Indeed, in order to be able to practice professionally as a nurse, a wide range of skills must be acquired in real environments so that the student can provide the patient with quality care (Bengtsson and Ohlsson, 2010; DeBourgh, 2012; Lewallen and DeBrew, 2012). Half of the education of nursing students in the European Union (EU) is conducted in clinical environments (Directive 2013/55/EU of the European Parliament of the Council, 2013). The quality of clinical environments and the competence of the clinical supervisor in the mentoring of nursing students (Jack et al., 2018; Pitkänen et al., 2018) substantially affects the learning of nurses (Pramila-Savukoski et al., 2020). The teaching in clinical practices involves student-centered learning, and not only in the knowledge that must be acquired (Bengtsson and Ohlsson, 2010). This new competency-based learning approach aims to enable nursing students to participate in self-directed learning (Lekan et al., 2011), maintaining fundamental and close communication with their clinical educators (Papathanasiou et al., 2014, Vizcaya-Moreno et al., 2018). Feedback (Almalkawi et al., 2018) and evaluation of students' learning by clinical supervisors is critical (Tuomikoski et al., 2019), so that they can apply their knowledge, skills and critical thinking (Haraldseid et al., 2015; Aktaş and Karabulut, 2016; Glynn et al., 2017).

This means new rules should be adopted so that the student and the teacher become agents who participate in the educational process. The supervision of practical training is increasingly important in the new nursing curricula for this reason. The interaction between the educator and the learner is a central element of the educational service, and one of the main indicators of satisfaction (Oldfield and Baron, 2000; Vizcaya-Moreno et al., 2018). Some studies report that the clinical education period is characterized by poor physical and mental health, a loss of interest in the profession and unacceptable care (Jeffries et al., 2013; Zamanzadeh et al., 2015; Drayton-Brooks et al., 2017). However, other studies mention positive experiences in the acquisition of knowledge, skills and attitudes of students in their clinical practices (Mulready-Shick and Flanagan, 2014; Nishioka et al., 2014; Glynn et al., 2017). The quality of clinical practices is a very significant influence on student's adherence, academic performance and closely related to their professional development of nursing competence (Perry et al., 2018). Based on previous literature, our hypothesis is as follows: (*H1) Higher mean student scores will predict higher levels of satisfaction with clinical practice in nursing students*.

This satisfaction of a nursing student during their clinical practice and their consequent involvement in the process can be influenced by many different factors, ranging from social and psychological factors to environmental and academic factors (El Ansari and Oskrochi, 2004). The literature includes studies that highlight the following factors in nursing students' satisfaction with their practices: the clinical educator, the learning environment, the activities carried out by the student and the organization of the practices (Milton-Wildey et al., 2014; Salamonson et al., 2015; Payne, 2016). These findings suggest the following hypothesis: *H2 Preference in the choice of the practice center, the distance to the practice center, the number of students assigned to the clinical educator, the type of service, the type of center and the type of management have an influence on nursing students' satisfaction with clinical practice*. Nursing students' satisfaction with their clinical practice helps students feel prepared for their future work, and is associated with their future work intentions (Milton-Wildey et al., 2014). Employment policies for recruiting and retaining nursing staff in hospitals should also be concerned with strategies to improve student education in their training (Barnett et al., 2010). Nurses are essential professionals in healthcare systems (Allen, 2018), and in order to prepare students adequately for the workplace and retain them in the workforce, the curricula of educational programs must succeed in providing students with the knowledge necessary to competently manage their role providing patient care in a difficult work environment (Hayes et al., 2006). These aspects are basic in any profession, but they are paramount for nurses because of the need for student nurses in order to offset the retirement of nurses (Aiken et al., 2009) and a serious shortage of nurses around the world (World Health Organization, 2020). In addition, the lack of job satisfaction among nurses has been associated with professional dissatisfaction as a powerful predictive factor for considering moving abroad (Ma et al., 2010), in search of better salaries, and a better quality of life (Granero-Lazaro et al., 2017).

This study focused on assessing how factors in the academic environment (preference in the choice of the practice center, the mean score of students, distance to the practice center, number of students assigned to the clinical educator, type of service, type of center and type of management) influence nursing students' satisfaction with their practice, using two different approaches: hierarchical regression models (HRM) and qualitative comparative analysis (QCA).

## Materials and Methods

### Participants

A cross-sectional analytical study was conducted in a convenience sample of 574 students on the nursing degree course at a private university in Valencia. Seventy-nine percentage (454) were women and the average age was (M = 24.4; SD = 6). See Table 1 for detailed information.

**Table 1:** Characteristics of the sample.

**Characteristics (*N* = 574)**	***N***	**%**
**Gender**		
Women	456	79
Men	118	21
**Academic year**		
Second	148	26
Third	418	71
Fourth	18	3
**Type of service**		
Hospitalization units	393	68
Special services	181	32
**Type of management**		
Public	347	60
Private	227	40
**Students working while studying**	159	28
**Students satisfied with their preference in the choice of the practice center**	499	87
**Type of center**		
Hospitals	416	72
Old people's homes	158	28

This study complied with the basic principles of the Helsinki Declaration (World Medical Association, 2013), with an emphasis on the anonymity of the data collected, confidentiality and non-discrimination of participants. This study was approved by the Research Ethics Committee of the University where the study was developed. All participants received detailed information about the objective and the procedure and were informed about confidentiality. The inclusion criteria were being a student on the clinical practice of the nursing degree course who gave their informed consent prior to their inclusion in the study. The anonymity and confidentiality of the information provided were respected. The data collection was carried out during the 2016/2017 academic year and consisted of completing an online questionnaire lasted about 20 min.

### Measures

The variables related to the service (a type of service, type of management and type of center), preference in the choice of the practice center, the mean score of students, distance to the practice center and the number of students assigned to the clinical educator.

The following validated instruments were used to measure the perceived satisfaction with the practices:

Clinical Learning Environment (CLE-1995; Dunn and Burnett, 1995), is a 23-item instrument composed of five subscales: staff-student relationships, preceptor's commitment, patient relationships, student satisfaction and hierarchy and ritual, rated on a 5-point Likert scale ranging between 1 (*strongly disagree*) and 5 (*strongly agree*). Examples of the items are: “I am happy with the experience I have had on this ward” “This was a good ward for my learning.” The reliability coefficients using Cronbach's alpha range from high (0.85) to marginal (0.63).

Clinical Learning Environment and Supervision (CLES-2002; Saarikoski and Leino-Kilpi, 2002), is a 27-item instrument divided into five sub-dimensions with the following number of items: ward atmosphere (five items); leadership style of the ward manager (four items); premises of nursing care on the ward (four items); premises of learning on the ward (six items) and supervisory relationship (eight items). The respondent answers the statements on a five-step Likert-type scale. The alternatives in the Likert scale were: (1) fully disagree; (2) disagree to some extent; (3) neither agree nor disagree; (4) agree to some extent and (5) fully agree. Examples of the items are: “The staff were easy to approach,” “There was a positive atmosphere on the ward.” The reliability coefficients of the sub-dimensions in this sample ranged from high (0.94) to marginal (0.73) using Cronbach's alpha.

Clinical Learning Environment Inventory (CLEI-2003; Chan, 2003). This instrument has 35 items, with 7 each items assessing five scales: personalization, student involvement, task orientation, innovation, and individualization, using a 4-point Likert-type scale with the alternatives of (1) strongly agree, (2) agree, (3) disagree, and (4) strongly disagree. Sample items include: “Students are generally allowed to work at their own pace,” “There are opportunities for students to express opinions in this ward.” Chan developed an instrument with two roles: for measuring the student's real perceived satisfaction with their practices, and for measuring the student's desired satisfaction with their practices. This aims to determine whether what the student wants is similar to their real experience. All the dimensions were Cronbach's Alpha >0.66, in both the real and in the desire instrument.

Clinical Learning environment, supervision and nurse teacher (CLES+T – 2008; Saarikoski et al., 2008). This consists of 34 items on five scales; supervisory relationship, pedagogical atmosphere on the ward, role of the nurse teacher, leadership style of the ward manager, and premises of nursing on the ward. The alternatives in the 5-step continuum scale (used in all phases of the study) were: (1) fully disagree, (2) disagree to some extent, (3) neither agree nor disagree, (4) agree to some extent, and (5) fully agree. Sample items included “In our common meetings I felt that we are colleagues,” and “The focus on the meetings was on my learning needs.” The reliability coefficients of the other sub-dimensions of CLES+T instrument using Cronbach's alpha ranged from high (0.96) to marginal (0.77). In this study the validated Spanish version was used (Vizcaya-Moreno et al., 2015). Cronbach's alpha was between 0.80 and 0.97.

Clinical Assessment Instrument (IEC-2009; Navarro, 2009), containing 67 items and five subscales in the Spanish language, 12 on program organization, 19 on teaching practice, 14 on student role, eight on student environment learning and eight on clinical experience. Each item was answered on a five-category Likert scale: 1 “strongly disagree,” 2 “partially disagree,” 3 “neither disagree nor agree,” 4 “partially agree” and 5 “strongly agree.” Sample items included “The objectives proposed were relevant to the achievement of learning,” and “Teachers facilitate students' clinical learning.” Cronbach's alpha was between 0.92 and 0.71.

To measure student participation in nursing activities during clinical practice (Slaughter-Smith et al., 2012; Zhang et al., 2012; Kristofferzon et al., 2013), the NIC taxonomy (NIC = Nursing Interventions Classification) (Ahn and Choi, 2015) was used. The instrument showed adequate psychometric properties: χ2/df = 1.36; NNFI = 0.97; CFI = 0.97; IFI = 0.97; RMSEA = 0.02 (IC = 0.018–0.025); α_total_ = 0.96; α_clinicaleducator_ = 0.97; α _learningenvironment_ = 0.94; α_activitiesperformed_ = 0.93; α _universityorganization_ = 0.92. It consisted of a 43-item separated into four dimensions of satisfaction (with the clinical educator, the activities performed, the learning environment and the university organization) and the average overall satisfaction for each of these dimensions. A 5-point Likert scale was chosen, where 1 was the equivalent to “totally disagree” and 5 to “totally agree.” Sample items included “Nursing professionals are interested in the supervision of students,” and “Working conditions were appropriate for learning.”

### Statistical Analyses

Descriptive analysis (mean, standard deviation) and hierarchical regression models (HRM) were used by means of the IBM SPSS Statistics 24 software package (IBM Corporation), while the qualitative comparative analysis (QCA) was carried out by means of fsQCA 2.5 (Claude and Christopher, 2014).

Linear regression methods compare the individual contribution of each variable to determine which one best explains the result. fsQCA is a variant of qualitative comparative analysis (QCA) and is based on defining the conditions that lead to the configurations causing the result of interest, expressing the combination rather than the individual contribution (Vis, 2012). The fundamental characteristics of this methodology are as follows (Ragin, 2008, 2014): (1) fsQCA studies are based on Boolean algebra, and its associated tools (e.g., truth table, consistency and coverage scores, set coincidence). (2) fsQCA is based on qualitative evidence using small or medium-sized samples, although although working with larger samples is possible (Vis, 2012); (3) fsQCA is based on the combination of variables, including equifinality, i.e., the different paths that lead a specific outcome; (4) fsQCA is used in regional analysis among other disciplines because of its advantages over correlation methods (García-Álvarez-Coque et al., 2017). This method has been developed to effectively approach research questions about which combination of conditions is associated with a particular result (Greckhamer et al., 2018).

The raw data was prepared according to the literature before performing the QCA (Ragin, 2008). First, all the missing values were eliminated and the variables recalibrated (Eng and Woodside, 2012). In our case (0 = woman;1 = man), (0 = second year; 0.49 = third year; 1 = fourth year), (0 = not working while studying; 1 = working while studying), (0 = specialized services; 1 = hospitalization), (0 = public institution; 1 = private institution), (0 = home care provider; 1 = hospitals), (0 = dissatisfied preference in the choice of the practice center; 1 = satisfied preference in the choice of the practice center) and all the dimensions of satisfaction were recoded (totality, clinical educator, learning environment, activities performed and organization of clinical practice) (0 = 3; 0.49 = 3.1 to 4; 1 = 4.1 to 5). The continuous variables, i.e., those related to the average student score, distance from the practice center and the number of students assigned to the clinical educator were recalibrated with fsQCA 2.5, by multiplying their components and then considering the three proposed thresholds (Woodside, 2013): 10% (low level or all outside the set), 50% (medium level, neither inside nor outside the set) and 90% (high level or all inside the set). The participants' responses to the analysis variables were coded as 0 and 1, where 0 means absence and 1 means presence. However, for continuous variables and interview factors composed of different elements, three values had to be taken into account to automatic recalibration. In the first (0), an observation with this value is completely outside the defined range (low level of agreement/value, in our case low satisfaction); the second (0.5) considers a mid-point, neither inside nor outside the framework (average level of agreement/value, average level of satisfaction); and the last (1) considers the observation completely within the framework (high level of agreement/value, high level of satisfaction) (Ragin, 2008).

The necessary and sufficient conditions were then used to assess the influence on the satisfaction of the variables associated with the service (type of service, type of management and type of center), preference in the choice of the practice center, average score of students, distance to the practice center and the number of students assigned to the clinical educator. The fsQCA analysis revealed three possible solutions for sufficient analysis: complex, parsimonious and intermediate (the latter was recommended by Ragin, 2008).

## Results

### Descriptive Statistics and Calibration Values

The most important descriptors and calibration values of the variables examined are shown in Table 2.

**Table 2:** Main descriptions and calibration values.

	**Number students/clinical educator period**	**Distance to the practice center**	**Students' mean score**
M	3.69	22.11	9.19
SD	5.27	14.36	0.65
Min	1	0	2.34
Max	43	60	10
**Calibration values**		
P10	1	5	8.50
P50	3	20	9.33
P90	6	40	9.73

*M, mean; SD, standard deviation; Min, minimum; Max, maximum; P10, 10th percentile; P50, 50th percentile; P90, 90th percentile*.

### Hierarchical Regression Models (HRM)

The variables studied were analyzed using hierarchical regression models. The criteria variables were satisfaction (total, clinical educator, activities performed, learning environment and university organization of the clinical practice). The independent variables were related to the service (type of service, type of management and type of center), preference in the choice of the practice center, students' mean score, distance to the practice center and number of students assigned to the clinical educator. First, the variables of preference in the choice of the practice center, students' mean score, distance to the practice center were performed, and the number of students per clinical educator period were then entered in a second step. Finally, the variables related to the service (type of service, type of management and type of center) were included in the third step.

The variables related to the preference in the choice of the practice center, students' mean score, distance to the practice center were included, and the number of students per clinical educator period (Table 3) was estimated for 5% of overall satisfaction (*R*^2^ adjusted = 0.05, *p* ≤ 0.001), 4% of clinical educator satisfaction (*R*^2^ adjusted = 0.04, *p* ≤ 0.001), 5% of environment satisfaction (*R*^2^ adjusted = 0.05, *p* ≤ 0.001) and finally 2% of activity satisfaction (*R*^2^ adjusted = 0.02, *p* ≤ 0.01).

**Table 3:** Hierarchical regression analysis with the satisfaction variable.

**Variable**	**Overall Satisfaction**		**Clinical educator satisfaction**		**Environment satisfaction**		**Activity satisfaction**		**Organization satisfaction**	
**Predictors**	**ΔR^**2**^**	**β**	**ΔR^**2**^**	**β**	**ΔR^**2**^**	**β**	**ΔR^**2**^**	**β**	**ΔR^**2**^**	**β**
Step 1	0.06^***^		0.05^***^		0.05^***^			0.03^**^		0.01^*^
Preference in the choice of the practice center		−0.04		−0.07		0.00		−0.03		−0.03
Students' mean score		0.23^***^		0.21^***^		0.22^***^		0.16^***^		0.12^**^
Distance to the practice center		−0.07		−0.08^*^		−0.06		−0.02		−0.03
Step 2	0.00		0.05		0.00			0.00		0.00
Preference in the choice of the practice center		−0.04		−0.06		0.00		−0.03		−0.02
Students' mean score		0.23^***^		0.21^***^		0.22^***^		0.16^***^		0.12^**^
Distance to the practice center		−0.07		−0.08		−0.06		−0.03		−0.03
Number of students per clinical educator period		−0.00		−0.03		0.00		0.07		−0.03
Step 3	0.00		0.05		0.00			0.00		0.01
Preference in the choice of the practice center		−0.05		−0.06		−0.00		−0.04		−0.03
Students' mean score		0.23^***^		0.21^***^		0.22^***^		0.16^***^		0.11^**^
Distance to the practice center		−0.06		−0.08		−0.06		−0.03		−0.01
Number of students per clinical educator period		−0.00		−0.03		0.00		0.07		−0.01
Type of service		0.03		−0.02		0.05		0.05		0.05
Type of management		−0.01		0.03		−0.03		−0.04		−0.05
Type of center		−0.01		0.00		0.03		0.02		−0.08
Total $R_{\text{adjusted}}^{2}$	0.05^***^		0.04^***^		0.05^***^			0.02^**^		0.01

* *p ≤ 0.05*;

** *p ≤ 0.01*;

*** *p ≤ 0.001*.

The linear regression model showed that the mean score of students determined a positive and significant beta coefficient in their satisfaction in overall terms (β = 0.23; *p* ≤ 0.001), and with their clinical educator (β = 0.21; *p* ≤ 0.001), environment (β = 0.22; *p* ≤ 0.001), activity (β = 0.16; *p* ≤ 0.001) and organization (β = 0.11; *p* ≤ 0.01).

### Fuzzy-Set Qualitative Comparative Analysis (fsQCA)

Necessary conditions are those that must always be present for a given outcome to occur, while sufficient conditions refer to those conditions (or a combination of them), that can lead to an outcome, even if they are not always present (Ragin, 2008).

#### Necessary Condition Analysis

The results obtained (Table 4), showed that it appears that there are no necessary conditions for satisfaction since the consistency is <0.90 in all cases (Ragin, 2008).

**Table 4:** Necessary condition analysis for satisfaction.

	**Overall satisfaction**		**Clinical educator satisfaction**		**Environment satisfaction**		**Activity satisfaction**		**Organization satisfaction**	
	**Cons^‡^**	**Cov^§^**	**Cons^‡^**	**Cov^§^**	**Cons^‡^**	**Cov^§^**	**Cons^‡^**	**Cov^§^**	**Cons^‡^**	**Cov^§^**
Preference in the choice of the practice center	0.87	0.90	0.86	0.84	0.87	0.91	0.87	0.90	0.87	0.92
High average score	0.58	0.95	0.58	0.89	0.57	0.95	0.57	0.95	0.56	0.94
Distance to the practice center	0.49	0.92	0.48	0.84	0.49	0.91	0.50	0.92	0.49	0.93
Number of students per clinical educator period	0.44	0.93	0.43	0.85	0.44	0.93	0.45	0.95	0.43	0.92
Hospitalization	0.69	0.91	0.68	0.84	0.69	0.92	0.69	0.92	0.69	0.93
Private management	0.72	0.90	0.72	0.83	0.73	0.91	0.73	0.91	0.72	0.91
Hospital	0.40	0.90	0.41	0.86	0.39	0.89	0.39	0.90	0.40	0.92

‡ *consistency*;

§ *coverage; condition needed: consistency ≥ 0.90*.

#### Sufficiency Analysis

A sufficiency analysis considers the solution coverage (variance explained, the number of observations that can be explained by a given combination of conditions) and the solution consistency (the reliability or relevance of the model). It also includes raw coverage (variance explained, how many cases or observations can be explained by a particular path or combination of conditions) and the unique coverage indicates the number of observations that can be clarified by one given path, but not by others (Ragin, 2008; Eng and Woodside, 2012).

Table 5 summarizes the three main sufficient conditions for the intermediate satisfaction solution according to the format Fiss (2011). In sufficiency analyses, a model is informative, when the consistency is above or around 0.75 (Eng and Woodside, 2012). When measuring overall satisfaction, 13 paths explained 74% of the high levels of overall satisfaction (overall consistency = 0.94; overall coverage = 0.74). The most important path for explaining it was the interaction of preference in the choice of the practice center, hospital and the proximity to the practice center (raw coverage = 0.33; consistency = 0.94), which accounted for 33% of cases with high overall satisfaction.

**Table 5:** Summary of the three main sufficient conditions for the intermediate solution of satisfaction.

**Frequency cutoff: 1**	**Overall satisfaction**			**Clinical educator satisfaction**			**Environment satisfaction**			**Activity satisfaction**			**Organization satisfaction**		
	**Consistency cut-off: 0.94**			**Consistency cut-off: 0.85**			**Consistency cut-off: 0.93**			**Consistency cut-off: 0.93**			**Consistency cut-off: 0.94**		
	**1**	**2**	**3**	**1**	**2**	**3**	**1**	**2**	**3**	**1**	**2**	**3**	**1**	**2**	**3**
Preference in the choice of the practice center	●	●	●			●	●		●		●	●		●	
High mean score						●	●				●		●		●
Distance to the practice center	○	●			●	●		●	○		●	○		●	
Number of students per clinical educator period				○	○			○	●		○	●	○		○
Hospitalization			○	●						●				●	●
Private management		○	○	●	○	○	○	○		○			○	○	○
Hospital	●	●	●	○	●	●	●	●	●	●	●	●	●	●	
Raw coverage	0.33	0.32	0.20	0.22	0.21	0.20	0.32	0.21	0.21	0.38	0.15	0.14	0.21	0.17	0.15
Unique coverage	0.03	0.04	0.05	0.08	0.04	0.04	0.07	0.03	0.02	0.24	0.03	0.03	0.03	0.05	0.01
Consistency	0.94	0.95	0.90	0.89	0.90	0.88	0.96	0.97	0.95	0.93	0.97	0.97	0.97	0.95	0.97
Overall solution consistency			**0.94**			**0.89**			**0.95**			**0.94**			**0.96**
Overall solution coverage			**0.74**			**0.73**			**0.65**			**0.70**			**0.62**

●, *presence of condition*; ○, *absence of condition*.

*Expected vector for Satisfaction (Overall, clinical educator, environment, activity and organization): 1,1,0,0,1,1,1 (0: absent; 1: present) according format Fiss (2011). Terms/values in bold, type information on the model as a whole considering all the conditions, not only the most important*.

Sixteen paths were found that explained 73% in the high levels of satisfaction with the clinical educator (overall consistency = 0.89; overall coverage = 0.73). The most important path that explains high levels of satisfaction with the clinical educator was the result of the interaction of hospitalization, private management, home care and low student numbers per clinical educator period, which accounted for 22% of the cases with high satisfaction with the clinical educator (raw coverage = 0.22; consistency = 0.89).

Twelve paths explained 65% of high levels of satisfaction with the learning environment (overall consistency = 0.95; overall coverage = 0.65). The most important path for explaining high levels of satisfaction with the learning environment was the interaction of preference in the choice of the practice center, students' high mean score, hospital and public management, which accounted for 32% of the cases (raw coverage = 0.32; consistency = 0.96).

When high levels of satisfaction with the activities performed were considered, 10 paths explained 70% (overall consistency = 0.94; overall coverage = 0.70). The most important path for explaining high levels of satisfaction with the activities performed arose from the combination of hospital, hospitalization and public management, which accounted for 38% of the cases (raw coverage = 0.38; consistency = 0.93).

Finally, 16 paths accounted for 62% in the high levels of satisfaction with the organization of the clinical practice (overall consistency = 0.96; overall coverage = 0.62). The most important path accounts for 21% of the cases (raw coverage = 0.21; consistency = 0.97), and this was the combination the hospital, the students' high mean score, a small number of students period/clinical educator, and public management.

## Discussion

This study extends the research on factors influencing nursing students' satisfaction in clinical practices where they learn important practical skills, their professional role, behavior, attitudes and values (Sandvik et al., 2014). The factors influencing the satisfaction with their clinical practice related to the academic environment (preference in the choice of the practice center, mean students' grade, distance to the practice center, number of students assigned to the clinical educator) and the service (type of service, type of center and type of management) were examined using complementary methodologies regression models and fsQCA models.

In general, the first hypothesis, i.e., higher mean student scores predict higher levels of satisfaction with clinical practices in nursing students, can be accepted in the regression model and partially accepted in fsQCA model. The regression models showed that the unique predictive variable is the students' mean score for all dimensions of satisfaction (overall satisfaction, with the clinical educator, the activities performed, the learning environment and the university organization). According to the literature, one of the most predictive indicators for measuring academic performance is the academic grade (Takashima et al., 2019). In the fsQCA model, the students' mean score is a condition for satisfaction with the learning environment and with the organization of the clinical practice. A quality learning environment and adequate organization of practices are considered key elements in the satisfaction of nursing students (Bisholt et al., 2014). For this reason, including student participation and perceptions in the evaluation of the quality of educational programs is today considered essential (Haraldseid et al., 2015), given that improving student experiences in their clinical learning environment can improve student learning outcomes and their satisfaction with the clinical practice (Pimparyon et al., 2000; Payne, 2016).

In the second hypothesis, the preference in the choice of the practice center, the distance to the practice center, the number of students assigned to the clinical educator, type of service, the type of center and the type of management have an influence on nursing students' satisfaction with clinical practices. This hypothesis can be accepted in the fsQCA model and partially accepted in the regression model. In general, fsQCA present higher levels of prediction than regression models. In the regression models, the variables related to the academic environment slightly estimated all the dimensions of satisfaction apart from university organization. The necessary conditions for satisfaction did not apply in the fsQCA models. In the sufficiency analysis, the most important conditions for predicting overall satisfaction were preference in the choice of the practice center, hospital and proximity to the practice center. For the clinical educator, the conditions were hospitalization, private management, home care and few students per clinical educator period. For satisfaction with the learning environment, the interactions were preference in the choice of the practice center, students' high mean score, hospital and public management. In the activities performed, the interactions were hospital, hospitalization and public management. Finally, in satisfaction with the clinical practice organization, the interactions were the hospital, students' high mean score, a low number of students period/clinical educator and public management. These results confirm that the number of students, type of center, management and service were conditions considered fundamental for the satisfaction of nursing students with the clinical educator and with the organization of the clinical practice by the university (Dunn and Burnett, 1995; Bergjan and Hertel, 2013; Ahn and Choi, 2015; Wafaa-Gameel et al., 2015). The service in which the clinical practice was carried out and the supervision of the clinical educator have a direct impact on the students' learning and satisfaction with the clinical educator and the organization of the clinical practice, the guidance and information (Milton-Wildey et al., 2014). Constructive feedback and reflective discussion from the clinical educator (Burden et al., 2018) improves the students' clinical knowledge and reasoning (McSharry and Lathlean, 2017). In that the student receives from the university, as part of the strategy to provide better support for students, influences the student's perception of the learning environment in the hospital (Chuan and Barnett, 2012; Lamont et al., 2015).

In addition, fsQCA models showed that the type of center (hospital) and public management were important conditions in satisfaction with the learning environment and with the activities. The type of service is a significant condition in satisfaction with the clinical educator and the activities. The literature indicates that human resources, materials, and even the way students are supervised are different in publicly managed facilities (O'Brien et al., 2019). Appropriate planning of the type of center, service, the students' clinical placement and students' clinical learning activities are important to ensure that the environment offers the student an appropriate learning situation (Sundler et al., 2014) and gives the student an overall perspective of nursing work and the various interventions that professionals may have to perform, from hospital to primary care (McInnes et al., 2015) so that the student has a comprehensive vision and education of nursing.

Despite the interest of the research, this study has some limitations. The study was conducted using a convenience sample at a single university, and as such the results cannot be generalized. It would be necessary to use a larger and more representative sample of other universities in future research. Another limitation of the study is the use of self-report measures, which can introduce social desirability biases. Using other heterocompleted measures and/or having objective external measurements would be necessary in future research.

As for implications for practice, this study provides information about how the interactions of conditions influence nursing students' satisfaction with clinical practice using fsQCA. There are no studies that examine the different academic variables that influence nursing students' satisfaction with clinical practice using this methodology. Likewise, the QCA models and linear models have different objectives and should be used in a complementary way (Ragin, 2008). In conclusion, the regression model shows the unique predictive variable is the students' mean score for all dimensions of satisfaction. In the fsQCA model, the students' mean score, the type of center (hospital), the type of management (public), the type of service, the preference in the choice of the practice center and the number of students were conditions considered fundamental for the satisfaction of nursing students with their clinical practice (with their educator, environment, activities and organization). These results can be considered on the interventions and practices programs related to the improvement of clinical practice. These measures will have a positive impact on student satisfaction and offers the student an appropriate learning situation (Sundler et al., 2014). They can be incorporated into university programs to improve the education of future professionals through clinical teaching models based collaboration between the university and the hospital to enhance evidence-based learning, thereby achieving significant changes in the quality of student education (Harvey et al., 2013). In addition, improved clinical practice planning enhances clinical educator attitudes (O'Brien et al., 2014).

A proper understanding of how the education of future nursing professionals is involved in the working relationship and the professional aspirations of students within the different roles in nursing could lead to appropriate strategies for creating more effective and robust collaboration between universities and hospitals (Limoges and Jagos, 2015). Appropriate education programmes and improved management in the recommended clinical settings are required to facilitate the transition of nurses from university to practice (Jeffries et al., 2013; Zamanzadeh et al., 2015; Drayton-Brooks et al., 2017), increase nurse retention (Ward and McComb, 2018), and positive attitudes. This study can be considered a first step toward the study of academic environmental factors that influence the satisfaction of nursing students with their practices.

## Data Availability Statement

The raw data supporting the conclusions of this article will be made available by the authors, without undue reservation.

## Ethics Statement

The studies involving human participants were reviewed and approved by Research Ethics Committee of the Catholic University of Valencia San Vicente Mártir UCV 2017-2018-032. The patients/participants provided their written informed consent to participate in this study.

## Author Contributions

DF-G, MCG-E, EC-R, and VP-G: made a substantial contribution to the concept or design of the work, or acquisition, analysis or interpretation of data, drafted the article or revised it critically for important intellectual content, approved the version to be published, and have participated sufficiently in the work to take public responsibility for appropriate portions of the content. All authors contributed to the article and approved the submitted version.

## Conflict of Interest

The authors declare that the research was conducted in the absence of any commercial or financial relationships that could be construed as a potential conflict of interest.

## References

[B1] Ahn Y. H. Choi J. (2015). Factors affecting Korean nursing student empowerment in clinical practice. Nurse Educ. Today 35, 1301–1306. 10.1016/j.nedt.2015.08.007 26323887

[B2] Aiken L. H. Cheung R. B. Olds D. M. (2009). Education policy initiatives to address the nurse shortage in the United States: in these economic times, it is shortsighted to allow attractive nursing jobs to go vacant when scores of prospective students are being turned away from nursing schools. Health Affairs 28, 646–656. 10.1377/hlthaff.28.4.w646 PMC2718732 19525285

[B3] Aktaş Y. Y. Karabulut N. (2016). A Survey on Turkish nursing students' perception of clinical learning environment and its association with academic motivation and clinical decision making. Nurse Educ. Today 36, 24–128. 10.1016/j.nedt.2015.08.015 26417713

[B5] Allen D. (2018). Translational mobilisation theory: A new paradigm for understanding the organizational elements of nursing work. Int. J. Nurs. Stud. 79, 36–42. 10.1016/j.ijnurstu.2017.10.010 29128687

[B6] Almalkawi I. Jester R. Terry L. (2018). Exploring mentors' interpretation of terminology and levels of competence when assessing nursing students: an integrative review. Nurse Educ. Today 69, 95–103. 10.1016/j.nedt.2018.07.003 30029042

[B7] Barnett T. Namasivayam P. Narudin D. A. A. (2010). A critical review of the nursing shortage in Malaysia. Int. Nurs. Rev. 57, 32–39. 10.1111/j.1466-7657.2009.00784.x 20487472

[B8] Bengtsson M. Ohlsson B. (2010). The nursing and medical students motivation to attain knowledge. Nurse Educ. Today 30, 150–156. 10.1016/j.nedt.2009.07.005 19692152

[B9] Bergjan M. Hertel F. (2013). Evaluating students' perception of their clinical placements — Testing the clinical learning environment and supervision and nurse teacher scale (CLES+T scale) in Germany. Nurse Educ. Today 33, 1393–1398. 10.1016/j.nedt.2012.11.002 23200088

[B10] Bisholt B. Ohlsson U. Engström A. K. Johansson A. S. Gustafsson M. (2014). Nursing students' assessment of the learning environment in different clinical settings. Nurse Educ. Pract. 14, 304–310. 10.1016/j.nepr.2013.11.005 24355802

[B11] Burden S. Topping A. E. O'Halloran C. (2018). Mentor judgements and decision-making in the assessment of student nurse competence in practice: a mixed-methods study. J. Adv. Nurs. 74, 1078–1089. 10.1111/jan.13508 29171082

[B12] Chan D. S. (2003). Validation of the clinical learning environment inventory. West. J. Nurs. Res. 25, 519–532. 10.1177/0193945903253161 12955969

[B13] Chuan O. L. Barnett T. (2012). Student, tutor and staff nurse perceptions of the clinical learning environment. Nurse Educ. Pract. 12, 192–197. 10.1016/j.nepr.2012.01.003 22277167

[B14] Claude R. Christopher R. (2014). Acq [Computer Programme], Version 2.1.12. Houston, TX: University of Houston-Downtown.

[B15] Cronin J. Taylor S. (1992). Measuring service quality: a reexamination and extension. J. Mark. 56, 55–68. 10.1177/002224299205600304

[B16] DeBourgh G. A. (2012). Synergy for patient safety and quality: academic and service partnerships to promote effective nurse education and clinical practice. J. Prof. Nurs. 28, 48–61. 10.1016/j.profnurs.2011.06.003 22261605

[B17] Directive 2013/55/EU of the European Parliament of the Council (2013). Official Journal of the European Union no L 354/132. Available online at: http://data.europa.eu/eli/dir/2013/55/oj (accessed November 04, 2020).

[B18] Drayton-Brooks S. M. Gray P. A. Turner N. P. Newland J. A. (2017). Building clinical education training capacity in nurse practitioner programs. J. Prof. Nurs. 33, 422–428. 10.1016/j.profnurs.2017.02.002 29157570

[B19] Dunn S. V. Burnett P. (1995). The development of a clinical learning environment scale. J. Adv. Nurs. 22, 1166–1173. 10.1111/j.1365-2648.1995.tb03119.x 8675872

[B20] El Ansari W. Oskrochi R. (2004). What ≪really≫ affects health professions students' satisfaction with their educational experience? Implications for practice and research. Nurse Educ. Today 24, 644–655. 10.1016/j.nedt.2004.09.002 15519448

[B21] Eng S. Woodside A. G. (2012). Configural analysis of the drinking man: fuzzy-set qualitative comparative analyses. Addict. Behav. 37, 541–543. 10.1016/j.addbeh.2011.11.034 22178601

[B22] Falk T. Hammerschmidt M. Schepers J. J. (2010). The service quality-satisfaction link revisited: exploring asymmetries and dynamics. J. Acad. Mark. Sci. 38, 288–302. 10.1007/s11747-009-0152-2

[B23] Fiss P. C. (2011). Building better causal theories: a fuzzy set approach to typologies in organization research. Acad. Manage. J. 54, 393–420. 10.5465/amj.2011.60263120

[B24] García-Álvarez-Coque J. M. Mas-Verdú F. Roig-Tierno N. (2017). Technological innovation versus non-technological innovation: different conditions in different regional contexts? Qual. Quant. 51, 1955–1967. 10.1007/s11135-016-0394-2

[B25] García-Pascual F. Prado-Gascó V. Alguacil M. Valantine I. Calabuig-Moreno F. (2020). Future intentions of fitness center customers: effect of emotions, perceived well-being and management variables. Front. Psychol. 11:2425 10.3389/fpsyg.2020.547846 PMC7545106 33101123

[B26] Glynn D. M. McVey C. Wendt J. Russell B. (2017). Dedicated educational nursing unit: clinical instructors role perceptions and learning needs. J. Prof. Nurs. 33, 108–112. 10.1016/j.profnurs.2016.08.005 28363384

[B27] Granero-Lazaro A. Blanch-Ribas J. M. Roldán-Merino J. F. Torralbas-mOrtega J. Escayola-Maranges A. M. (2017). Crisis in the health sector: Impact on nurses? Working conditions. Enferm. Clin. 27, 163–171. 10.1016/j.enfcle.2017.03.004 28408097

[B28] Greckhamer T. Furnari S. Fiss P. C. Aguilera R. V. (2018). Studying configurations with qualitative comparative analysis: best practices in strategy and organization research. Strateg. Organ 16, 482–495. 10.1177/1476127018786487

[B29] Green D. (1994). What Is Quality in Higher Education? London: Society for Research into Higher Education, Ltd.

[B30] Haraldseid C. Friberg F. Aase K. (2015). Nursing students' perceptions of factors influencing their learning environment in a clinical skills laboratory: a qualitative study. Nurse Educ. Today 35, e1–e6. 10.1016/j.nedt.2015.03.015 25873478

[B31] Harvey T. Calleja P. Thi D. P. (2013). Improving access to quality clinical nurse teaching—A partnership between Australia and Vietnam. Nurse Educ. Today 33, 671–676. 10.1016/j.nedt.2012.02.001 22381381

[B32] Hayes L. J. O'Brien-Pallas L. Duffield C. Shamian J. Buchan J. Hughes F.. (2006). Nurse turnover: a literature review. Int. J. Nurs. Stud. 43, 237–263. 10.1016/j.ijnurstu.2005.02.007 15878771

[B33] Jack K. Hamshire C. Harris W. E. Langan M. Barrett N. Wibberley C. (2018). “My mentor didn't speak to me for the first four weeks”: perceived unfairness experienced by nursing students in clinical practice settings. J. Clin. Nurs. 27, 929–938. 10.1111/jocn.14015 28815761

[B34] Jeffries P. R. Rose L. Belcher A. E. Dang D. Hochuli J. F. Fleischmann D.. (2013). A clinical academic practice partnership: a clinical education redesign. J. Prof. Nurs. 29, 128–136. 10.1016/j.profnurs.2012.04.013 23706965

[B35] Kristofferzon M. Mårtensson G. Mamhidir A. Löfmark A. (2013). Nursing students' perceptions of clinical supervision: The contributions of preceptors, head preceptors and clinical lecturers. Nurse Educ. Today 33, 1252–1257. 10.1016/j.nedt.2012.08.017 22995594

[B36] Lamont S. Brunero S. Woods K. P. (2015). Satisfaction with clinical placement – The perspective of nursing students from multiple universities. Collegian 22, 125–133. 10.1016/j.colegn.2013.12.005 26285417

[B37] Lekan D. A. Corazzini K. N. Gilliss C. L. Bailey D. E. (2011). Clinical leadership development in accelerated baccalaureate nursing students: an education innovation. J. Prof. Nurs. 27, 202–214. 10.1016/j.profnurs.2011.03.002 21767817

[B38] Lewallen L. P. DeBrew J. K. (2012). Successful and unsuccessful clinical nursing students. J. Nurs. Educ. 51, 389–395. 10.3928/01484834-20120427-01 22533497

[B39] Limoges J. Jagos K. (2015). The influences of nursing education on the socialization and professional working relationships of Canadian practical and degree nursing students: a critical analysis. Nurse Educ. Today 35, 1023–1027. 10.1016/j.nedt.2015.07.018 26260523

[B40] Löfmark A. Thorkildsen K. Råholm M. B. Natvig G. K. (2012). Nursing students' satisfaction with supervision from preceptors and teachers during clinical practice. Nurse Educ. Pract. 12, 164–169. 10.1016/j.nepr.2011.12.005 22225731

[B41] Ma A. X. Quinn Griffin M. T. Capitulo K. L. Fitzpatrick J. J. (2010). Demands of immigration among Chinese immigrant nurses. Int. J. Nurs. Pract. 16, 443–453. 10.1111/j.1440-172X.2010.01868.x 20854341

[B42] McInnes S. Peters K. Hardy J. Halcomb E. (2015). Clinical placements in Australian general practice: (Part 1) the experiences of pre-registration nursing students. Nurse Educ. Pract. 15, 437–442. 10.1016/j.nepr.2015.04.003 25979152

[B43] McSharry E. Lathlean J. (2017). Clinical teaching and learning within a preceptorship model in an acute care hospital in Ireland; a qualitative study. Nurse Educ. Today 51, 73–80. 10.1016/j.nedt.2017.01.007 28130976

[B44] Milton-Wildey K. Kenny P. Parmenter G. Hall J. (2014). Educational preparation for clinical nursing: the satisfaction of students and new graduates from two Australian universities. Nurse Educ. Today 34, 648–654. 10.1016/j.nedt.2013.07.004 23880324

[B45] Moreno F. C. Prado-Gasco V. Hervas J. C. Nunez-Pomar J. Sanz V. A. (2015). Spectator emotions: effects on quality, satisfaction, value, and future intentions. J. Bus. Res. 68, 1445–1449. 10.1016/j.jbusres.2015.01.031

[B46] Mulready-Shick J. Flanagan K. (2014). Building the evidence for dedicated education unit sustainability and partnership success. Nurs. Educ. Perspect. 35, 287–293. 10.5480/14-1379 25291923

[B47] Navarro N. (2009). Diseño y validación de un instrumento de evaluación clínica (Design and validation of a clinical evaluation instrument). Rev. Educ. Ciencias Salud 6, 79–86.

[B48] Nishioka V. Coe M. Hanita M. Moscato S. (2014). Dedicated education unit: nurse perspectives on their clinical teaching role. Nurs. Educ. Perspect. 35, 294–300. 10.5480/14-1381 25291924

[B49] O'Brien A. Giles M. Dempsey S. Lynne S. McGregor M. E. Kable A. (2014). Evaluating the preceptor role for pre-registration nursing and midwifery student clinical education. Nurse Educ. Today 34, 19–24. 10.1016/j.nedt.2013.03.015 23623277

[B50] O'Brien A. T. Mcneil K. Dawson A. (2019). The student experience of clinical supervision across health disciplines–Perspectives and remedies to enhance clinical placement. Nurse Educ. Pract. 34, 48–55. 10.1016/j.nepr.2018.11.006 30458410

[B51] Oldfield B. M. Baron S. (2000). Student perceptions of service quality in a UK university business and management faculty. Qual. Assur. Educ. 8, 85–95. 10.1108/09684880010325600

[B52] Olorunniwo F. Hsu M. K. Udo G. J. (2006). Service Quality, customer satisfaction, and behavioral intentions in the service factory. J. Serv. Mark. 20, 59–72. 10.1108/08876040610646581

[B53] Papathanasiou I. V. Tsaras K. Sarafis P. (2014). Views and perceptions of nursing students on their clinical learning environment: teaching and learning. Nurse Educ. Today 34, 57–60. 10.1016/j.nedt.2013.02.007 23481172

[B54] Parasuraman A. Zeithalm V. Berry L. (1985). A Conceptual model of service quality and its implications for future research. J. Mark. 49, 41–50. 10.1177/002224298504900403

[B55] Parasuraman A. Zeithalm V. Berry L. (1988). SERVQUAL: a multiple-item scale for measuring consumer perceptions of service quality. J. Retail. 64, 12–40.

[B56] Payne C. (2016). Transitions into practice: first patient care experiences of baccalaureate nursing students. Nurse Educ. Today 16, 251–257. 10.1016/j.nepr.2015.09.011 26546235

[B57] Perry C. Henderson A. Grealish L. (2018). The behaviours of nurses that increase student accountability for learning in clinical practice: an integrative review. Nurse Educ. Today 65, 177–186. 10.1016/j.nedt.2018.02.029 29587209

[B58] Pimparyon S. M. Caleer S. Pemba S. Roff P. (2000). Educational environment, student approaches to learning and academic achievement in a Thai nursing school. Med. Teach. 22, 359–364. 10.1080/014215900409456

[B59] Pitkänen S. Kääriäinen M. Oikarainen A. Tuomikoski A. Elo S. Ruotsalainen H.. (2018). Healthcare students' evaluation of the clinical learning environment and supervision – a cross-sectional study. Nurse Educ. Today 62, 143–149. 10.1016/j.nedt.2018.01.005 29353088

[B60] Pramila-Savukoski S. Juntunen J. Tuomikoski A. M. Käriäinen M. Tomietto M. Kaučič B. M.. (2020). Mentors' self-assessed competence in mentoring nursing students in clinical practice: a systematic review of quantitative studies. J. Clin. Nurs. 29, 684–705. 10.1111/jocn.15127 31794105

[B61] Pucciarelli F. Kaplan A. (2016). Competition and strategy in higher education: managing complexity and uncertainty. Bus. Horiz. 59, 311–320. 10.1016/j.bushor.2016.01.003

[B62] Ragin C. C. (2008). Redesigning Social Inquiry: Fuzzy Sets and Beyond. Chicago: The University of Chicago Press.

[B63] Ragin C. C. (2014). The Comparative Method: Moving Beyond Qualitative and Quantitative Strategies. Berkeley, CA: University of California Press.

[B64] Rauyruen P. Miller K. E. (2007). Relationship quality as a predictor of B2B customer loyalty. J. Bus. Res. 60, 21–31. 10.1016/j.jbusres.2005.11.006

[B65] Saarikoski M. Isoaho H. Warne T. Leino-Kilpi H. (2008). The nurse teacher in clinical practice: developing the new sub-dimension to the clinical learning environment and supervision (CLES) scale. Int. J. Nurs. Stud. 45, 1233–1237. 10.1016/j.ijnurstu.2007.07.009 17803996

[B66] Saarikoski M. Leino-Kilpi H. (2002). The clinical learning environment and supervision by staff nurses: developing the instrument. Int. J. Nurs. Stud. 39, 259-267. 10.1016/S0020-7489(01)00031-1 11864649

[B67] Salamonson Y. Everett B. Halcomb E. Hutchinson M. Jackson D. Mannix J.. (2015). Unravelling the complexities of nursing students' feedback on the clinical learning environment: a mixed methods approach. Nurse Educ. Today 35, 206–211. 10.1016/j.nedt.2014.08.005 25200510

[B68] Sandvik A. Eriksson K. Hilli Y. (2014). Becoming a caring nurse –A Nordic study on students' learning and development in clinical education. Nurse Educ Pract. 14, 286–292. 10.1016/j.nepr.2013.11.001 24290731

[B69] Slaughter-Smith C. Helms J. E. Burris R. (2012). Nursing staff perceptions of student contributions in clinical settings. J. Nurs. Educ. 51, 54–57. 10.3928/01484834-20111130-02 22132717

[B70] Sundler A. J. Björk M. Bisholt B. Ohlsson U. Engström A. K. Gustafsson M. (2014). Student nurses' experiences of the clinical learning environment in relation to the organization of supervision: a questionnaire survey. Nurse Educ. Today 34, 661–666. 10.1016/j.nedt.2013.06.023 23850574

[B71] Takashima M. Burmeister E. Ossenberg C. Henderson A. (2019). Assessment of the clinical performance of nursing students in the workplace: exploring the role of benchmarking using the Australian Nursing Standards Assessment Tool (ANSAT). Collegian 26, 502–506. 10.1016/j.colegn.2019.01.005

[B72] Tuomikoski A. Ruotsalainen H. Mikkonen K. Kääriäinen M. (2019). Nurses' experiences of their competence at mentoring nursing students: a systematic review of qualitative studies. Nurse Educ. Today 85:104258. 10.1016/j.nedt.2019.104258 31830638

[B73] Vis B. (2012). The Comparative advantages of fsQCA and regression analysis for moderately Large-N analyses. Sociol. Methodol. 41, 168–198. 10.1177/0049124112442142

[B74] Vizcaya-Moreno M. F. Pérez-Cañaveras R. M. De Juan J. Saarikoski M. (2015). Development and psychometric testing of the clinical learning environment, supervision and nurse teacher evaluation scale (CLES+ T): the Spanish version. Int. J. Nurs. Stud. 52, 361–367. 10.1016/j.ijnurstu.2014.08.008 25220932

[B75] Vizcaya-Moreno M. F. Pérez-Cañaveras R. M. Jiménez-Ruiz I. de Juan J. (2018). Student nurse perceptions of supervision and clinical learning environment: a phenomenological research study. Enfermeria Glob. 17, 319–331. 10.6018/eglobal.17.3.276101

[B83] Wafaa-Gameel A. EL-Banan S. H. A. Al-Seraty W. H (2015). Effective clinical learning environment as perceived by nursing students at AL dawadmi, applied medical sciences college: actual versus preferred characteristics. Int. J. Nurs. Educ. 5, 1–6. 10.15520/ijnd.2015.vol5.iss05.94.01-06

[B76] Ward A. E. McComb S. A. (2018). Formalising the precepting process: a concept analysis of preceptorship. J. Clin. Nurs. 27, 873–881. 10.1111/jocn.14203 29193513

[B77] Woodside A. G. (2013). Moving beyond multiple regression analysis to algorithms: calling for adoption of a paradigm shift from symmetric to asymmetric thinking in data analysis and crafting theory. J. Bus. Res. 66, 463–472. 10.1016/j.jbusres.2012.12.021

[B78] World Health Organization (2020). State of the World's Nursing 2020: Investing in Education, Jobs and Leadership. Licence: CC BY-NC-SA 3.0 IG. Retrieved from: http://apps.who.int/iris (accessed November 02, 2020).

[B79] World Medical Association (2013). Ethical principles for medical research involving human subjects, in 64th WMA General Assembly October 2013, Fortaleza, Brazil.

[B80] Zamanzadeh V. Jasemi M. Valizadeh L. Keogh B. Taleghani F. (2015). Lack of preparation: Iranian nurses' experiences during transition from college to clinical practice. J. Prof. Nurs. 31, 365–373. 10.1016/j.profnurs.2015.01.005 26194969

[B81] Zeithaml V. A. Berry L. L. Parasuraman A. (1993). The nature and determinants of customer expectations of service. J. Acad. Mark. Sci. 21, 1–12. 10.1177/009207039321100110557397

[B82] Zhang Q. Zeng T. Chen Y. Li X. (2012). Assisting undergraduate nursing students to learn evidence-based practice through self-directed learning and workshop strategies during clinical practicum. Nurse Educ. Today 32, 570–575. 10.1016/j.nedt.2011.05.018 21664015

